# Prevalence and clinical and psychological correlates of high fear of cancer recurrence in patients newly diagnosed with head and neck cancer

**DOI:** 10.1002/hed.25812

**Published:** 2019-06-07

**Authors:** Spela Mirosevic, Belinda Thewes, Carla van Herpen, Johannes Kaanders, Thijs Merkx, Gerry Humphris, Robert J. Baatenburg de Jong, Johannes A. Langendijk, C. René Leemans, Chris H. J. Terhaard, Irma M. Verdonck‐de Leeuw, Robert Takes, Judith Prins, the NET‐QUBIC Consortium

**Affiliations:** ^1^ Department of Family Medicine Medical Faculty Ljubljana Ljubljana Slovenia; ^2^ Department of Medical Psychology Radboud University Medical Centre Nijmegen The Netherlands; ^3^ Department of Medical Oncology Radboud University Medical Centre Nijmegen The Netherlands; ^4^ Department of Radiation Oncology Radboud University Medical Centre Nijmegen The Netherlands; ^5^ Department Maxillofacial Surgery Radboud University Medical Centre Nijmegen The Netherlands; ^6^ School of Medicine University of St. Andrews St. Andrews Scotland, UK; ^7^ Department of Otolaryngology and Head and Neck Surgery Erasmus Cancer Institute, Erasmus MC Rotterdam The Netherlands; ^8^ Department of Radiation Oncology University of Groningen, University Medical Centre Groningen Groningen The Netherlands; ^9^ Department of Otolaryngology‐Head and Neck Surgery VU University Medical Center Amsterdam The Netherlands; ^10^ Department of Radiotherapy University Medical Center Utrecht The Netherlands; ^11^ Department of Clinical Psychology Vrije Universiteit Amsterdam The Netherlands; ^12^ Department of Head and Neck Oncology Radboud University Medical Centre Nijmegen The Netherlands; ^13^ Project Kubus, Vumc, Afdeling KNO Amsterdam The Netherlands

**Keywords:** anxiety, depression, fear of recurrence, head and neck cancer, smoking

## Abstract

**Background:**

Patients with head and neck cancer (HNC) are vulnerable to fear of cancer recurrence (FCR) and psychiatric morbidity. We investigated the prevalence of high FCR and demographic, clinical, psychological, and psychiatric factors associated with high FCR prior to the start of the treatment.

**Methods:**

In a cross‐sectional substudy of the large ongoing prospective NET‐QUBIC study questionnaires and psychiatric interviews of 216 patients newly diagnosed with HNC were analyzed.

**Results:**

High FCR was observed in 52.8% of patients and among those 21.1% also had a lifetime history of selected anxiety or major depressive disorder. FCR was not related to any clinical characteristics; however, younger age, higher anxiety symptoms, introversion, greater needs for support regarding sexuality, and being an exsmoker were significantly associated with higher FCR.

**Conclusion:**

Factors associated with high FCR provide us with a better conceptual understanding of FCR in patients newly diagnosed with HNC.

## 1 INTRODUCTION

Head and neck cancer (HNC) includes a range of tumors that arise in oral cavity, pharynx, larynx, nasal cavity, paranasal sinuses, and salivary glands. Each year, HNC accounts for more than 550 000 new diagnoses and 380 000 deaths worldwide.^1^ In the Netherlands, about 3000 people are diagnosed with HCN annually.^2^ Past research demonstrates that patients with HNC are particularly vulnerable to develop fear of cancer recurrence (FCR), defined as “fear, worry, or concern relating to the possibility that cancer will come back or progress,”^3^ with between 31% and 83% of patients reporting elevated levels of FCR.^4^, ^5^, ^6^, ^7^, ^8^ Moreover, FCR has been consistently reported as one of the most prevalent unmet needs for help of cancer survivors in various cancer types^9^, ^10^, ^11^ and one of the most common concerns that patients with HNC wish to discuss in specialist consultations.^12^

The model of Lee‐Jones et al^13^ offers a good theoretical basis for understanding patients' reactions and fears about the possibility of cancer progression or cancer coming back. This model proposes that patients' fear might be triggered by cognition and emotions caused by internal cues (eg, physical symptoms) and/or external cues (eg, doctors' appointments). Fear can further be caused by specific behaviors such as seeking advice at health professionals and personal checking behavior or psychological effects such as misinterpretation of symptoms or an increase in somatic anxiety. Patients with HNC within 5 years have a significant chance to relapse.^14^ Patients newly diagnosed with HNC have frequent medical consultations and exams in the diagnosis phase and extensive treatment involving higher level of physical symptoms (eg, pain, sore dry mouth, and throat),^15^ providing an understandable basis for FCR.

However, similar to studies involving patients with other cancer types, most longitudinal and cross‐sectional studies in patients with HNC fail to find an association between FCR and objective indices of poor prognosis (eg, tumor site and tumor stage).^6^, ^7^, ^12^, ^16^, ^17^ Treatment‐, symptom‐, and patient‐related factors appear to be more important determinants of FCR. Although there is some evidence on various factors associated with FCR in HNC, most of these findings were derived from patients in the active treatment or posttreatment survivorship phase and less is known on how these factors are associated in the patients newly diagnosed with HNC. Albeit patients newly diagnosed with cancer might be more fearful about progression rather than recurrence, the definition of FCR encompasses both the possibility that cancer will progress as well as that it might come back.^3^ Moreover, high levels of FCR have been identified as soon as prior to the treatment,^18^ which points out that worries about recurrence/progression can occur early in the cancer trajectory.

Previous studies involving patients with HNC suggest a significant relationship between FCR and younger age, having chemotherapy or radiotherapy, physical symptoms (eg, pain), disfigurement, psychological factors (including symptoms of anxiety and depression), and lifestyle factors (eg, smoking).^4^, ^6^, ^7^, ^12^, ^16^, ^19^ High levels of FCR have been consistently associated with poorer QoL in patients with HNC.^4^, ^16^, ^20^ However, it is noteworthy that the majority of studies to date on FCR and various variables in HNC are cross‐sectional and involve relatively small samples. Moreover, there is currently no evidence on how FCR and personality, fatigue, and cancer‐specific distress (eg, intrusion and avoidance) are connected in patients with HNC; however, outside of HNC, studies with other types of patients with cancer suggest that higher levels of intrusive stress symptoms, neuroticism, and fatigue are related to higher FCR.^21^, ^22^, ^23^ There are currently no studies addressing the association between FCR and unmet needs in patients with HNC; however, a study of mixed cancer survivors indicated that higher FCR was associated with all five unmet supportive care needs, including needs regarding health system and information, sexuality, patient care, physical and daily living, and psychological needs.^24^

Many previous studies among patients with HNC showed a relationship between FCR and psychological symptoms (eg, anxiety and depression).^5^, ^6^, ^7^, ^12^, ^19^ Psychological distress is usually assessed by self‐reported questionnaires, mostly the Hospital Anxiety and Depression Scale (HADS), which is a frequently used screening tool in HNC research for detecting symptoms of anxiety and depression.^25^, ^26^ However, there is a lack of consensus on an optimal cutoff score for identifying probable clinical cases of anxiety and depression (the cutoff score ranges from a low of 5 to high of 11).^25^ The golden standard for identifying psychiatric morbidity is considered to be psychiatric interviews. Several studies in patients with various cancer types have explored the relationship between FCR and psychiatric disorders and showed that the presence of anxiety disorders^21^, ^27^ and post‐traumatic stress disorder (PTSD) symptoms,^28^ was positively associated with FCR. Simard et al^22^ found that as FCR severity increases, intrusive thoughts appear more like obsessions (as seen in the obsessive‐compulsive disorder). However, studies have found that only 16%‐43% of patients with high FCR have a comorbid psychiatric disorder,^21^, ^27^, ^29^, ^30^, ^31^, ^32^, ^33^ suggesting that FCR is an independent entity that needs to be studied as a separate construct. Importantly, none of those reported studies focused specifically on patients with HNC. Data suggest that patients with HNC report the second highest prevalence of any mental disorders among all cancer types, with a 4‐week DSM‐IV overall disorder prevalence of 41% and 12‐month prevalence of 39%.^34^, ^35^ Therefore, understanding the relationship between FCR and psychiatric morbidity in this cancer patient subgroup is of particular importance.

HNC, its recurrence, and mortality are strongly associated with environmental and lifestyle risk factors like tobacco and excessive alcohol consumption.^36^ Although few studies have investigated the relationship between FCR and tobacco use, those conducted to date reported a positive association between high FCR and increased tobacco use,^20^ and that exsmokers with higher FCR were more likely to relapse by 12 months follow‐up.^37^ Similarly, successful smoking cessation might also be related to reduced FCR, with one prospective study of 73 patients with HNC showing that those who quit smoking during the first 15 months after diagnosis had significantly less FCR compared with those who continued to smoke or who relapsed.^19^ Further research is needed to understand this complex relationship between smoking and FCR in patients newly diagnosed with HNC.

The prevalence rate of elevated FCR among patients with HNC is reported to be high (>30%),^4^, ^5^, ^6^, ^7^, ^8^ however only two studies have been conducted in patients newly diagnosed with HNC, with varying estimates ranging from 31%^5^ to 62% using varying measures of FCR.^8^ Importantly, only a study from Savard and Ivers reported a validated cutoff score of 13.^8^ In accordance with Lee‐Jones's FCR model, the presence of physical symptoms associated with HNC and the frequency of medical consultation and exams in the diagnosis phase could lead to a higher FCR in this population. Due to the FCR's negative impact on well‐being, there is a growing need to identify factors associated with high FCR in HNC, especially in patients newly diagnosed with HNC, so that appropriate supportive care interventions can be designed and offered to those patients with HNC who need it. Therefore, the purpose of this study was to explore the prevalence of FCR and the strength of association between FCR and psychiatric disorders (selected anxiety and major depressive disorder), and demographic, clinical, psychological, and behavioral features associated with FCR in a representative cohort of newly diagnosed Dutch patients with HNC. The current study was exploratory and variables introduced above were chosen based on factors associated with high FCR in the past literature.^17^ Therefore, the objective of this study was to: (a) explore the prevalence and psychiatric morbidity of patients with HNC experiencing high FCR and (b) assess the potential factors associated with FCR in patients with HNC.

## 2 PATIENTS AND METHODS

### 2.1 Patients and procedure

This study was conducted using baseline data (before start of the treatment) of the first 254 patients newly diagnosed with HNC from a large ongoing prospective cohort study investigating the long‐term course of quality of life (QoL) in patients with HNC and their caregivers (NET‐QUBIC study; www.kubusproject.nl). The present sample consisted of only patients newly diagnosed with HNC who completed baseline assessment of FCR (N = 216).

Study participants were recruited between March 2014 and July 2016 in the five medical centers in the Netherlands (VU University Medical Centre Amsterdam [VUmc], University Medical Center Groningen, Radboud University Medical Center Nijmegen, University Medical Center Utrecht, and Erasmus Medical Center Rotterdam). Every new patient with HNC was screened for eligibility. Eligible patients were 18 years or older, newly diagnosed with HNC (oral, oropharynx, hypopharynx, larynx, unknown primary; all stages), previously untreated and with plans for treatment with curative intent according to standard treatment guidelines, and able to write, read, and speak fluent Dutch. Exclusion criteria were malignancies of the salivary glands, nasopharyngeal malignancies, lymphoma, skin malignancies, thyroid cancer, and patients with severe psychiatric comorbidities which could impair the ability to give informed consent (eg, schizophrenia, Korsakoff's syndrome, and severe dementia). Patients with severe psychiatric difficulties were excluded based on the professional expertise of the physician or nurse involved in the recruitment of patients, and in consultation with the treating physician. Eligible patients were invited to participate by the treating surgeon, and the research physician provided them with more information about the study and with written information. All patients that agreed to participate signed a written consent. Ethical approval was obtained by the coordinating center (METc VUmc 2013.301), and local approval was obtained individually with each medical center. A more detailed explanation about the procedure and recruitment is explained elsewhere.^38^

### 2.2 Methods

Assessments were conducted at the hospital and/or the patient's home. Assessments included patient reported outcomes, interviews, and medical examinations.^38^

*Fear of recurrence* was measured using the Cancer Worry Scale (CWS), which is a reliable and valid self‐reported 8‐item scale.^39^ It measures worry about the risk of cancer recurrence and its impact on daily functioning. Total score ranges from 8 to 32. Higher scores indicate higher FCR. A cutoff score of 13 vs 14 (low FCR: ≤13, high FCR: ≥14) has been established to differentiate between those with normal FCR from those with bothersome levels of FCR (henceforth referred to as high FCR).^39^ This cutoff has been validated relative to the Fear of Cancer Recurrence Inventory cutoff score, which is validated measure.^40^ The Dutch version of the CWS is reliable (Cronbach's α = .87 and .89) and has been validated in various cancer populations.^40^, ^41^ Internal consistency in the present sample was high (Cronbach's α = 0.90; 95% confidence interval = 0.88, 0.92).

*Demographic and clinical characteristics* were collected in self‐report questionnaires and medical records. Demographic characteristics included age, sex, education, and living arrangement (coded as alone or cohabiting). Disease‐related characteristics included tumor location, tumor stage (from 0 to II coded as early and from III to IVB as late), time since diagnosis (measured in days), and World Health Organization performance status (0, able to carry all normal activity without restriction; 1, restricted in physical strenuous activity but ambulatory and able to carry out light work; 2, ambulatory and capable of all self‐care but unable to carry out any work). Data for physical comorbidity were assessed by the Adult Comorbidity Evaluation‐27 Index,^42^ which is a validated comorbidity instrument with a 27 items and 12 categories (eg, cardiovascular and body weight). For the analysis, only item severity was used. This item is originally coded as none, mild, moderate, and severe; however, we have categorized it as none‐mild or moderate‐severe.

*Substance use regarding alcohol and nicotine* was assessed using study‐specific items. Alcohol was analyzed as a continuous variable (number of alcohol beverages on a typical day) and smoking status was coded as smoking at the time of assessment (yes/no) or in the past (no, but I smoked in the past).

*Psychiatric morbidity* represented by the presence of lifetime depressive (major depressive disorder) and selected anxiety (panic disorder, social phobia, generalized anxiety disorder [GAD], and agoraphobia) disorders was assessed with the Composite International Diagnostic Interview (CIDI), which is based on the DSM‐IV criteria. Although the CIDI can assess current and lifetime disorders, only lifetime disorders were assessed for this study. The lifetime CIDI allowed for the determination of the history, recency (ie, within last week and more than 1 year ago), duration, and age of onset of episodes of selected anxiety and major depressive disorder.^43^ Fieldworkers from different backgrounds (eg, nurse, dietician, psychologist, and laboratory technician) were trained to conduct the CIDI in a standardized way. Additionally, all CIDI interviews were recorded and the coordinating field worker randomly checked and supervised for their quality. Patients were coded into the two groups: (a) no CIDI lifetime anxiety or major depressive disorder and (b) CIDI lifetime anxiety or major depressive disorder.

*Cancer‐specific distress* was assessed by the Impact of Event Scale‐Revised, which is a 22‐items scale^44^ and includes subscales of intrusion, sleep disturbance, hyperarousal, avoidance, and numbing. Items are scored on a 5‐point scale, ranging from 0 (“not at all”) to 4 (“extremely”). For the analysis, only subscale scores for intrusion and avoidance were used (8 items for avoidance and 7 items for intrusion), as those two appear to be most commonly associated with high FCR.^45^ The scale is well validated in the Netherlands, and Cronbach's alpha in the Dutch patients with cancer was reported to be .85 for avoidance and .92 for intrusion.^46^

*Psychological distress* expressed in symptoms of anxiety and depression was assessed with the HADS,^47^ which includes 14 items that are divided into two subscales: depression and anxiety. The maximum score on each subscale is 21 and higher score suggests elevated symptomatology. Internal consistency in the Dutch patients with cancer showed an adequate value (α coefficient was .89 for anxiety and .78 for depression).^46^ We have analyzed only scores for anxiety and depression and not the total distress scores.

QoL was assessed using 2 of the 6 functional scales from the core module of the EORTC QoL questionnaire (EORTC QLQ‐C30). Based on a team discussion and literature review, only physical functioning and global QoL subscale scores were used.^40^ A Dutch version tested in patients with cancer showed a Cronbach's α = .80 for physical functioning and .86 for global QoL.^48^, ^49^

*Supportive care needs* were assessed using the Supportive Care Needs Survey Short‐Form 34 (SCNS‐SF34), which is a 34 item measure that evaluates patient's need for supportive care with respect to physical and daily living, psychological, sexuality, patient care and support, and health system and information needs.^50^ In addition, a 1‐item specific HNC issues subscale was used from the SCNS‐HNC, which assesses problems such as chewing/swallowing, speech, and hearing.^51^ Lifestyle subscale was not included in the analysis, because we had assessed drinking and smoking problems separately (see demographic and clinical characteristics above). Evidence supports the validity and reliability of the Dutch versions of the SCNS‐SF34 (α = .79‐.95) and SCNS‐HNC (α = .89 for specific HNC issues).^51^

*Personality factors* were assessed using the FIVE Factor Inventory,^52^ which measures five dimensions of personality: neuroticism, extraversion, openness to experience, agreeableness, and conscientiousness. Each dimension consists of 12 items. In the Dutch patients with cancer, Cronbach's alpha showed a satisfactory result (.73‐.86).^53^ Based on a team discussion and literature review, only neuroticism and extraversion were included.

*Physical symptoms and functioning* were assessed with the following measures. For *fatigue*, Multidimensional Fatigue Inventory^54^ was used, which is a 20‐item self‐reported measure covering various dimensions of fatigue (ie, general, physical, mental fatigue, reduced motivation, and activity). For the analysis, the total score was used. Cronbach's alpha in the Dutch patients with cancer was reportedly adequate (.84). *Physical activity* was assessed with the Physical Activity Scale for the Elderly, which is a 10‐item questionnaire that measures activity levels over a timeframe of 1 week, ranging from 0 to 739 (higher score indicating greater physical activity).^55^ Finally, *pain* was measured by the Brief Pain Inventory (BPI), which assesses the types of pain, pain history, intensity, location, quality, and the degree of pain interference with activities of daily life.^56^ For the analysis with FCR, only one question was selected from the BPI (the mean severity pain).

### 2.3 Statistical analysis

Data were analyzed using SPSS version 22. Scores on self‐reported questionnaires and missing values were treated according to their manuals. The primary outcome was CWS score. Data on CIDI were analyzed only for the bivariate comparison of mean CWS with the CIDI lifetime anxiety or depressive disorder and were not included in the multivariate analysis.

Responders and nonresponders to the CWS were compared on age, clinical variables, anxiety and depression symptoms, and sex to examine whether there were significant differences between groups (Table 1). After eliminating CWS nonresponders from the analysis, descriptive statistics were used to explore the distribution of the data and to report the prevalence of high FCR (≥14) and the relationship between CIDI psychiatric morbidity and high FCR.

**Table 1:** Clinical and demographic characteristics

Characteristics	CWS completers^a^	CWS noncompleters^b^	*P* value^c^
No. of patients	216	38	
Age at diagnosis (mean ± SD, range)	62.3 ± 9.7, 37‐85	60.7 ± 10.8, 41‐84	.36
Sex (n, %)			
Female	66 (30.6)	6 (15.8)	.06
Male	150 (69.4)	32 (84.2)	
Living arrangement (n, %)			
Alone	40 (18.5)	13 (34.2)	.12
Cohabiting	143 (66.2)	21 (55.3)	
Missing	33 (15.3)	4 (10.5)	
Educational status (n, %)			
Primary education	10 (4.6)	3 (7.9)	.98
Lower or preparatory vocational education	45 (20.8)	8 (21.1)	
Intermediary general secondary education	27 (12.5)	6 (15.8)	
Senior general secondary education	34 (15.7)	6 (15.8)	
Higher general secondary education	19 (8.8)	4 (10.4)	
Higher professional education	33 (15.3)	5 (13.2)	
University	14 (6.5)	2 (5.3)	
Missing	34 (15.7)	4 (10.5)	
Smoking status (n, %)			.88
Regular smoker	55 (25.5)	1 (2.6)	
Occasional smoker	11 (5.1)	0	
Smoker in the past	113 (52.3)	5 (13.2)	
Never smoked	34 (15.7)	1 (2.6)	
Missing	3 (1.4)	31 (81.6)	
Alcohol (no. of beverages on a typical day)			.99
M ± SD, range	3.0 ± 3.8, 0‐28	3.0 ± 1.4, 0‐4	
Missing (n, %)	23 (10.6)	31 (81.6)	
Time since diagnosis (days) (n, %)	21.3 (18.6)	13 (9.0)	.24
Missing (n, %)	3 (1.4)	31(81.6)	
Tumor site (n, %)			.50
Oral cavity	65 (30.1)	7 (18.4)	
Oropharynx	74 (34.3)	15 (39.5)	
Hypopharynx	18 (8.3)	5 (13.2)	
Larynx	55 (25.5)	11 (28.9)	
Unknown primary	4 (1.9)	0	
Tumor stage (n, %)			.42
Early stage	89 (41.2)	13 (32.2)	
Late stage	127 (58.8)	25 (65.8)	
WHO performance status (n, %)			.79
0^d^	153 (74.3)	28 (73.7)	
1^e^	53 (24.8)	8 (21.1)	
3^f^	8 (3.7)	2 (5.3)	
ACE‐27 comorbidity scale (n, %)			.21
None‐mild	133 (61.6)	26 (68.4)	
Moderate‐severe	70 (32.4)	8 (21.1)	
Missing	13 (6.0)	4 (10.5)	
PASE, physical activity (M ± SD)	101 ± 82.8	84.8 ± 40.6	.64
Missing (n, %)	1 (0.5)	32 (84.2)	
BPI, mean severity pain (M ± SD)	4 ± 2	4.1 ± 2.0	.95
Missing (n, %)	135 (62.5)	22 (57.9)	
MFI, general fatigue (M ± SD)	10.9 ± 4.8	8.4 ± 2.4	.04
Missing (n, %)	4 (1.9)	31 (81.6)	
HADS, anxiety (M ± SD)	6.0 ± 4.0	6.8 ± 4.7	.63
Missing (n, %)	11 (5.1)	32 (84.2)	
HADS, depression (M ± SD)	4.3 ± 3.6	5.7 ± 5.8	.59
Missing (n, %)	9 (4.2)	32 (84.2)	
IES‐R intrusion (M ± SD)	4.9 ± 4.9	8.7 ± 5.2	.05
Missing (n, %)	8 (3.7)	31 (81.6)	
IES‐R avoidance (M ± SD)	3.8 ± 4.2	7.6 ± 4.2	.02
Missing (n, %)	12 (5.6)	31 (81.6)	
EORTC, physical functioning (M ± SD)	58.6 ± 18.4	97.9 ± 5.3	.001
Missing (n, %)	9 (4.2)	32 (84.2)	
EORTC, global quality of life (M ± SD)	68.9 ± 18.7	79.0 ± 6.7	.01
Missing (n, %)	9 (4.2)	32 (84.2)	
NEO‐FFI, neuroticism (M ± SD)	28.3 ± 7.5	28.1 ± 2.5	.89
Missing (n, %)	3 (1.4)	31 (81.6)	
NEO‐FFI, extraversion (M ± SD)	39.9 ± 6.9	38.0 ± 3.8	.48
Missing (n, %)	3 (1.4)	31 (81.6)	
SCNS‐SF34, physical and daily living (M ± SD)	17.5 ± 23.1	23.6 ± 31.1	.50
Missing (n, %)	9 (4.2)	31 (81.6)	
SCNS‐SF34, psychological (M ± SD)	28.0 ± 24.6	36.1 ± 29.7	.40
Missing (n, %)	9 (4.2)	31 (81.6)	
SCNS‐SF34, sexuality (M ± SD)	12.9 ± 20.4	10.7 ± 18.5	.78
Missing (n, %)	10 (4.6)	31 (81.6)	
SCNS‐SF34, health system, information, and patients support (M ± SD)	30.1 ± 21.6	26.4 ± 22.2	.66
Missing (n, %)	10 (4.6)	31 (81.6)	
SCNS‐HNC, head and neck cancer‐specific functioning (M ± SD)	15.4 ± 17.0	7.6 ± 15.0	.23
Missing (n, %)	10 (4.6)	31 (81.6)	

Abbreviations: ACE‐27, Adult Comorbidity Evaluation‐27 Index; BPI, Brief Pain Inventory; CWS, Cancer Worry Scale; FCR, fear of cancer recurrence; HADS, Hospital Anxiety and Depression Scale, IES‐R, Impact of Event Scale‐Revised; MFI, Multidimensional Fatigue Inventory; NEO‐FFI, FIVE Factor Inventory; PASE, Physical Activity Scale for the Elderly; SCNS‐HNC, Supportive Care Needs Survey‐HNC; SCNS‐SF34, Supportive Care Needs Survey Short‐Form 34; WHO, World Health Organization.

a Patients who completed primary outcome.

b Patients who did not complete the primary outcome.

c Based on *t* tests (for continuous variables) and chi‐square or Fischer test (for categorical variables).

d Able to carry out all normal activity without restriction.

e Restricted in physical strenuous activity but ambulatory, able to carry out light work.

f Ambulatory and capable of all self‐care but unable to carry out any work.

The relationship between CIDI psychiatric morbidity and FCR was calculated by comparing mean CWS scores between no CIDI lifetime selected anxiety or major depressive disorder and CIDI lifetime selected anxiety or major depressive disorder.

The distribution of CWS scores and residuals were examined for normality. After confirming that CWS scores were normally distributed, age‐adjusted univariate linear regression was used to explore the association between potential predictor variables and FCR. Univariate tests were age‐adjusted because younger age has been shown to be the most consistent predictor of elevated FCR.^45^ In the multivariate models, tolerance statistics and variance inflation factor of each potential predictor and the correlations among the predictors were examined to identify and address potential multicollinearity issues in the model. Potential predictors (ie, those significant in univariate tests at the *P* < .2 level) were entered into stepwise linear regression models, first sociodemographic, then clinical, psychological, and behavioral characteristics. Variables were retained in the penultimate model if they were significant at the *P* < .1 level. A final linear regression model was then used to examine the unique effects of modifiable psychological and behavioral characteristics on FCR when controlling for all previously significant clinical and demographics factors in addition to age. Variables in the final model were considered significant if *P* < .05.

## 3 RESULTS

The response rate of the baseline early release NET‐QUBIC data is 39%. Therefore, 254 baseline questionnaires were received. However, as FCR was our main outcome, 38 patients (15%) that did not complete the CWS or had too many missing items to calculate a total score of CWS (Table 1) were excluded from the analysis, resulting in a sample of 216 patients for the present analysis. A higher proportion of males (84.8% vs 69.4%, *P* = .06) and those living alone (34.3% vs 18.5, *P* = .12) completed the CWS, but these differences were not statistically significant (Table 1). Furthermore, there were no statistically significant differences found between completers and noncompleters of the CWS on other sociodemographic, clinical, or psychological characteristics.

### 3.1 Patients characteristics

Sociodemographic and clinical characteristics of the sample are shown in Table 1. Patients' mean age was 62.3 years (SD = 9.7), 69.4% of the sample represented males, and 66.2% of the patients were cohabiting with a partner or other person. Patients were diagnosed with oropharynx (34%), oral cavity (30%), larynx (26%), or hypopharynx (8%), and the majority were diagnosed in the late stage of the disease (59%). The majority (71.6%) had a good performance status (ie, were able to carry out normal activity) and 34.5% had moderate to severe comorbidity at the time of the study, which appears to be high.^57^

### 3.2 Prevalence of FCR and its severity

High FCR (ie, CWS score ≥ 14) was reported by 114 (52.8%) patients. Mean CWS score was 14.25 (SD = 4.6) for the entire sample, 17.7 (3.6) for participants with high FCR, and 10.5 (1.7) for those with low FCR.

### 3.3 Stepwise regression model: variables associated with FCR

Results of age‐adjusted univariate regressions are presented in Table 2. Briefly, when controlling for age, patients with HNC with higher FCR were more likely to be exsmokers, have restricted physical strenuous activity, worse QoL (only global QoL), higher neuroticism and introversion, and higher level of unmet needs in each of the five unmet need domains. Patients with HNC with higher FCR were also more likely to report higher levels of cancer‐specific intrusion and avoidance symptoms, higher symptoms of depression and anxiety, higher mean pain severity, and higher fatigue. There were no significant associations with self‐reported physical activity, or with any of the demographic or clinical variables assessed.

**Table 2:** Results of age‐adjusted associations of all potential predictors with FCR score (CWS‐total) for patients newly diagnosed with HNC (n = 216)

Variable	Beta	*t*	*P*
Sex	.337	0.502	.62
Education (dummy coded, “Primary” set as a reference variable)			.18 (overall model)
Primary (ref)			
Lower or preparatory vocational	.159	0.161	.87
Intermediary general secondary	−.276	−0.246	.81
Senior general secondary	−.625	−0.595	.55
Higher general secondary	−.182	−0.145	.88
Higher professional	.754	0.710	.48
University	−.429	−0.305	.76
Living arrangement			
Alone			
Cohabiting	−.443	−0.524	.60
Smoking (dummy coded, Yes, I smoke every day set as a reference variable)			.008 (overall model)
No, but I smoked in the past (ref)			
No	−.126	−1.803	.07
Yes, I smoke every day	−.071	−1.009	.31
Alcohol (no. of beverages on a typical day)	−.028	−0.317	.75
Time since diagnosis (days)	−.221	−1.161	.25
Tumor site (dummy coded, “Oropharynx” set as a reference variable)			.07 (overall model)
Oropharynx (ref)			
Oral cavity	.635	0.821	.41
Hypopharynx	1.053	0.878	.38
Larynx	.961	1.178	.24
Unknown primary	1.064	0.455	.65
Tumor stage			
Early			
Late	.301	0.476	.63
EORTC, physical functioning	−.033	−1.865	.06
EORTC, global quality of life	−.077	−4.735	<.001
NEO‐FFI, neuroticism	.230	5.969	<.001
NEO‐FFI, extraversion	−.159	−3.517	.001
SCNS‐SF34, physical and daily living	.031	2.374	.02
SCNS‐SF34, psychological	.084	7.367	<.001
SCNS‐SF34, sexuality	.058	3.973	<.001
SCNS‐SF34, health system, information, and patients support	.051	3.851	<.001
SCNS‐HNC, Head and neck cancer‐specific functioning	.047	2.743	.007
PASE physical activity	.002	0.525	.60
IES‐R intrusion	.327	5.510	<.001
IES‐R avoidance	.369	5.220	<.001
HADS, depression	.541	6.751	<.001
HADS, anxiety	.734	11.777	<.001
BPI, mean pain severity	.389	2.748	.007
WHO performance (dummy coded, “Able to carry” set as a reference)			.01 (overall model)
Able to carry all normal activity without restriction (ref)			
Restricted in physical strenuous activity but ambulatory and able to carry out light work	1.195	1.654	.10
Ambulatory and capable of all self‐care but unable to carry out any work	.532	0.325	.75
Comorbidity			
None‐mild			
Moderate‐severe	.703	1.026	.31
MFI general fatigue	.207	3.294	.001

Abbreviations: BPI, Brief Pain Inventory; CWS, Cancer Worry Scale; FCR, fear of cancer recurrence; HADS, Hospital Anxiety and Depression Scale, HNC, head and neck cancer; IES‐R, Impact of Event Scale‐Revised; MFI, Multidimensional Fatigue Inventory; NEO‐FFI, FIVE Factor Inventory; PASE, Physical Activity Scale for the Elderly; SCNS‐HNC, Supportive Care Needs Survey‐HNC; SCNS‐SF34, Supportive Care Needs Survey Short‐Form 34; WHO, World Health Organization.

Significant predictors in age‐adjusted analyses were selected for further examination in multivariate models. The results of the final model are shown in Table 3. Higher FCR levels were significantly associated with younger age (β = .203, *P* < .001), introversion (β = .115, *P* < .05), more symptoms of anxiety (β = .578, *P* < .001), having higher levels of unmet needs for help with sexual issues (β = .182, *P* = .001) and being an exsmoker (β = .130, *P* < .05). The model was found to be statistically significant (*F* [7, 185] = 24.724, *P* < .001) and explained 46.4% of the variance in FCR in patients with HNC. The strongest predictors of higher FCR were higher anxiety (HADS‐A) scores and younger age.

**Table 3:** Linear regression model predicting FCR scores

Predictors	Beta	*t*	*P*
Age	−.203	−3.692	>.001
HADS, anxiety	.578	10.477	>.001
NEO‐FFI, introversion	.115	2.040	.04
SCNS‐SF34, sexuality	.182	3.357	.001
Smoking status			
Regular smoker (ref)			
Occasional smoker	.014	0.252	.88
Smoker in the past	.130	2.038	.04
Never smoked	.009	0.146	.80

*Notes*. R = 0.695; R^2^ = 0.483; adjusted R^2^ = 0.464; SE of the estimate = 3.297. Beta is standardized regression coefficient.

Abbreviations: FCR, fear of cancer recurrence; HADS, Hospital Anxiety and Depression Scale, NEO‐FFI, FIVE Factor Inventory; SCNS‐SF34, Supportive Care Needs Survey Short‐Form 34.

### 3.4 Prevalence of selected lifetime psychiatric disorders and overlap with high FCR

The analysis of lifetime psychiatric disorders is based on 178 patients for whom data on CIDI anxiety disorders (including GAD, social phobia, panic disorder, and agoraphobia) and major depressive disorder and CWS scores were available. Patients for whom selected psychiatric disorders were available did not differ from patients for whom selected disorders were not available on any of the demographic or clinical characteristics (data not shown). The prevalence of selected lifetime CIDI anxiety disorders was 8.4% (n = 15), and 11.2% (n = 20) for CIDI major depressive disorder (see the Methods section for subtypes), however as some patients were found to have both (combined CIDI), the prevalence of having a lifetime history of either a lifetime selected anxiety or major depressive disorder was 16.3% (n = 29). Table 4 presents the group differences in means for CWS for patients with (a) no CIDI diagnosis vs (b) those diagnosed with selected CIDI anxiety disorders or major depression. Patients with a lifetime history of either selected anxiety or major depressive disorder reported significantly higher FCR than those without a diagnosis (*F* = .768, *P* = .006).

**Table 4:** Comparison of mean (SD) CWS for patients with (a) no CIDI lifetime anxiety or major depressive disorder vs (b) CIDI lifetime anxiety or major depressive disorder (N = 178)

Measure	No CIDI anxiety or major depressive disorder (n = 143)	CIDI anxiety or major depressive disorder (n = 29)	*P* value
CWS (M, SD)	13.73 (4.39)	16.34 (5.60)	.006

Abbreviations: CWS, Cancer Worry Scale; CIDI, Composite International Diagnostic Interview.

**Figure 1:**
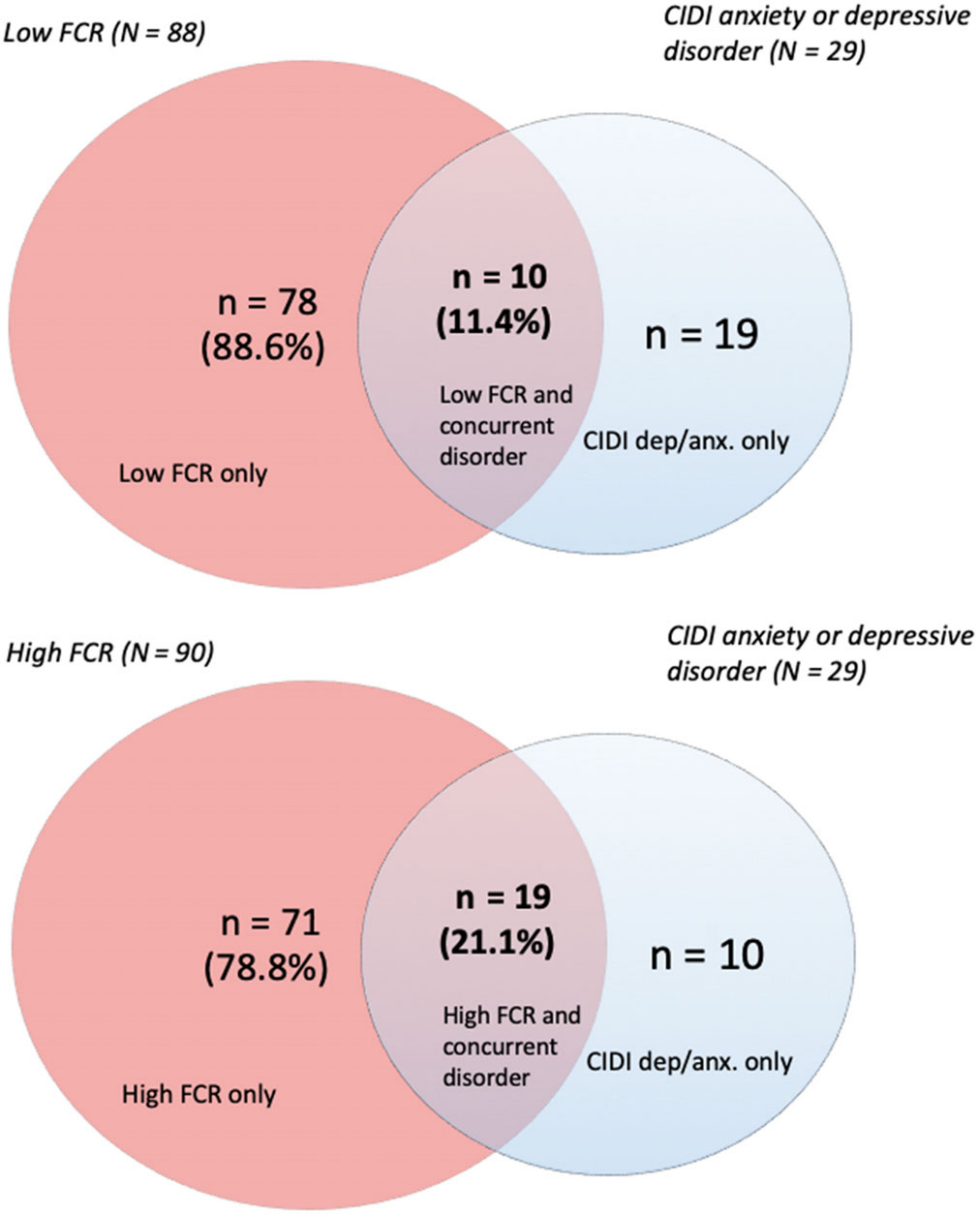
Lifetime psychiatric morbidity and low/high fear of cancer recurrence (FCR) in patients who completed the Composite International Diagnostic Interview (CIDI) interviews (n = 178) [Color figure can be viewed at wileyonlinelibrary.com]

Figure 1 illustrates the percentage of patients with low FCR and lifetime selected anxiety or major depressive disorder, and patients with high FCR and concurrent selected psychiatric disorders. Among patients with high FCR and for whom data for selected psychiatric morbidity was available (n = 90), 21.1% (n = 19) of the patients had a lifetime selected anxiety or major depressive disorder, which means that almost 80% of the patients with high FCR did not have lifetime selected psychiatric disorders. In the group of low FCR, there were 10 out of 29 patients (34%) with concurrent selected psychiatric disorder (Figure 1). There were no statistically significant differences found between the proportion of patients with high vs low FCR (21.1% vs 11.4%; *P* = .08) reporting lifetime selected psychiatric morbidity.

## 4 DISCUSSION

This study reports baseline data from the NET‐QUBIC study, an ongoing prospective population‐based cohort study in patients newly diagnosed with HNC. Approximately half (52%) of the patients reported high FCR. Being younger, more introverted, having higher levels of unmet needs for help with sexual issues, higher symptoms of anxiety, and being an exsmoker were identified as factors associated with high FCR. Patients with a lifetime history of selected anxiety disorders and major depressive disorder reported significantly higher levels of FCR (severity) than those without it; however, it is noteworthy that the majority of patients with high FCR did not additionally meet that criteria for a lifetime history of a selected anxiety or major depressive disorder, suggesting that while FCR may be associated with psychopathology, it is a distinct problem for the majority of HNC and therefore warrants specific attention.

The prevalence of high FCR in patients newly diagnosed with HNC in the present study is consistent with past literature in patients newly diagnosed with HNC, which reports prevalence rates ranging widely from 31%^5^ to 62%.^8^ However, two previous studies have used a variety of instruments to measure FCR and a variety of definitions of high FCR, therefore drawing comparisons with these studies is difficult. The high prevalence found in our study may, in part, be explained by the fact that our sample involved patients newly diagnosed with HNC who are unaware of the severity of their diagnosis, which may increase feelings of uncertainty. Although future longitudinal assessments will further screen how the prevalence changes through time, a prospective study by Savard and Ivers reported that a small sample of patients with HNC presented a stable pattern of elevated FCR across all six time points during the 18‐month follow‐up (60%‐66%).^8^

Several demographic and psychological characteristics emerged as factors associated with FCR. Like previous literature the present study found an association between high FCR and younger age.^4^, ^6^, ^7^, ^12^ With respect to psychological characteristics (symptoms of anxiety and depression, as measured with HADS), only symptoms of anxiety were found to be a significant predictor in the final model. This is in accordance with previous studies that used multivariate analysis and observed no significant association with depression.^17^, ^45^ One plausible explanation would be that anxiety is a concept different but more closely associated with FCR and particularly with a concept like cancer worry. However, these relationships may vary over time, and thus this possibility will be further monitored by the ongoing NET‐QUBIC study.

The present study also found that FCR is higher in more introverted patients. This novel finding might be explained by the fact that people high on introversion are less likely to seek social support and help of others when needed.^58^ Our results showed that neuroticism was not statistically associated with FCR in the multivariate models. Furthermore, other known predictors of high FCR such as intrusive and avoidant stress symptoms and poor QoL were also not significantly associated with higher FCR in the multivariate model in the present baseline analysis, but will be interesting to screen over time in future assessments.

An interesting and novel finding was the association between high FCR and unmet supportive care needs concerning sexuality. Our findings suggest that patients more fearful of recurrence also have higher levels of unmet needs for sexual care; but not on other domains of the supportive care unmet needs. A cross‐sectional study of mixed cancer groups (including HNC) on average 12 months after the diagnosis found high FCR to be associated not only with unmet needs for sexual care, but also with unmet needs with regard to health system and information, and patient care.^24^ Our sample consisted of patients newly diagnosed with HNC who have just entered the health system, which could to some degree explain the lack of these findings in our results. Although we cannot determine direction of the relationship of the unmet needs on sexual care and high FCR in our sample, the latter study hypothesized that FCR might interfere with patients' ability to relax, which might distract them and cause problems in their intimate relationships.^24^ Another plausible explanation would be that FCR affects couples,^59^ and communication difficulties can arise when both partners are greatly concerned but not communicating about their fears. In a couple dyad with high FCR, partners may feel distanced and isolated from one another in their intimate relationships.

This study provides further evidence of the relationship between high FCR and tobacco use. These data partly confirm findings from two other studies of patients with HNC concerning the relationship between smoking behavior and high FCR.^19^, ^37^ In our study, high FCR was associated only with smoking in the past but not current smoking. One plausible reason could be that exsmokers are more aware of the relationship between smoking and cancer, hence their efforts to quit. However, having higher FCR 3 months after initial treatment has been found to predict smoking or relapse to smoking at 12 months after surgery in previous research,^19^ raising the question whether high FCR predisposes exsmokers to smoking relapse if the problem is not adequately addressed. Future data analyses might examine whether exsmokers with high FCR are more likely to relapse to smoking than exsmokers with lower levels of FCR.

The results suggested significant associations between FCR and current self‐reported anxiety symptoms, as well as with prior lifetime history of selected anxiety disorders and major depressive disorder. The majority of patients with high FCR did not additionally have lifetime selected anxiety and major depressive disorders, and there was no statistical difference found between proportion of patients with high vs low FCR reporting selected lifetime psychiatric morbidity. This is somewhat consistent with previous research which reports a 16%‐43% overlap of FCR scores with screening instruments of psychiatric diagnoses (including GAD and hypochondriasis)^31^, ^32^ and 7%‐40% overlap with structured clinical interviews, depending on the type of psychiatric morbidity assessed.^30^, ^31^ However, none of these studies focused specifically on patients with HNC. Thus, data of the present study at least to some extent provide further evidence that FCR is a problem which is distinct from anxiety and depressive disorders for the majority of patients with cancer.^31^, ^32^ However, it is noteworthy that only lifetime CIDI was assessed and that patients were screened only for some types of anxiety disorder (see the Methods section) and major depressive disorder, therefore it is not known to what extent high FCR is related to other psychiatric disorders (substance dependence, personality disorders, dysthymic disorder, adjustment disorders, and PTSD) in this sample. Importantly, patients with severe psychiatric disorders were excluded from the study (see inclusion/exclusion criteria). By contrast, when examining psychological symptoms, like previous studies, the present study found a significant association between high FCR and HADS anxiety symptoms in the final multivariate model. Importantly, the analysis involved current difficulties as opposed to a prior lifetime history of difficulties, and therefore offers further evidence that there is indeed a meaningful overlap between these variables. High FCR might therefore be a prodromal indicator of a subsequent anxiety (or major depressive) disorder (and vice versa, ie, post‐diagnosis anxiety or depression as a precursor to high FCR); however, this hypothesis can be examined in future waves of data collection within this prospective study.

Some strengths and limitations of the study warrant consideration when interpreting the results. One of the strength of the study was that the data derived from a multicenter recruitment. Moreover, the present study included a relatively large and homogenous sample in terms of time of assessment and phase of treatment. However, homogenous characteristics of patients could limit the generalizability of the results. Another limitation is that only lifetime history of selected anxiety disorders and major depressive disorder was assessed and not current disorders and other psychiatric disorders. Moreover, the present findings are based on cross‐sectional baseline data precluding drawing conclusions about the causal or temporal direction of relationships between higher FCR and the factors associated with high FCR. Furthermore, due to the large number of analyses and the exploratory nature of the study, further data are needed to confirm these results. Future data collection in the NET‐QUBIC project will be used to replicate these findings, examine the course of high FCR during the first 5 years after diagnosis, and generate a better understanding of vulnerability factors for high FCR in patients with HNC. Moreover, in future, wave treatment will have started so we can look at the impact of treatment and its side effects on FCR.

It should be noted that one of the questions of the CWS includes an item relating to the worry about the heredity risk of cancer, and so it is difficult to consider it for HNC population. It is worth mentioning that the item content of the CWS relates directly only to the cancer recurrence and not progression, however the definition of the FCR covers both. Moreover, this is a longitudinal study that will follow patients with HNC through the years, and thus, for some patients, fear of progression will become fear of recurrence over time.

Albeit time since diagnosis was known, time of completing the questionnaires and interviews in relation to the multidisciplinary treatment planning meeting (in which prognosis is often discussed) was not known from the existing data set. This is relevant because it is not known to what extent patients were fully aware of their objective risk of recurrence at the time of completing the baseline questionnaire and interviews. Future NET‐QUBIC reports will also report on the association between treatment type and FCR, since two recent meta‐analyses reported that both chemotherapy and radiotherapy are significant predictors of high FCR,^60^, ^61^ although none of analyses were specifically made on patients with HNC. Another interesting question that will be explored in emerging prospective data is how FCR and cancer survival are related. Previous studies have only reported about the potential relationship between more global psychological distress (mainly depression) and disease progression/recurrence and mortality.^62^ Literature reviews suggest a small/moderate effect of psychological distress on cancer prognosis,^63^ however no data to this date are available on the relationship between FCR and survival.

In conclusion, approximately one in two of all patients newly diagnosed with HNC had FCR levels above the validated cutoff for high FCR shortly after diagnosis. As previously reported in other studies,^45^ FCR was not associated with tumor‐related characteristics, however higher FCR were found to be associated with being younger, more introverted, having higher symptoms of anxiety, more unmet needs for help with sexuality issues, and being an exsmoker.

For clinicians working with patients with HNC, these data help to raise awareness of the high prevalence of FCR in patients newly diagnosed with HNC. High FCR can sometimes be associated with hypervigilance for symptoms, overuse of medical testing, and/or avoidance of recommended medical investigations.^13^ A better understanding and detection of high FCR might encourage clinicians to tell patients about this and promote more appropriate follow‐up and cancer surveillance in vulnerable individuals. Brief screening tools for FCR including some specifically developed for the HNC setting (eg, Patients Concern Inventory; and a single‐item screener)^64^ are available and may assist clinicians to identify patients with HNC with high FCR who might benefit from new evidence‐based psychological interventions^65^, ^66^ for severe and disabling FCR.
